# Diagnosis and Management of Bacterial Meningitis by Pediatric Residents in Sudan

**DOI:** 10.7759/cureus.101622

**Published:** 2026-01-15

**Authors:** Razan M Hassan, Ahmed Ali, Galal Eldin E Babekir

**Affiliations:** 1 Children’s Emergency Department, Barking, Havering and Redbridge University Hospitals NHS Trust, London, GBR; 2 Pediatrics Department, Faculty of Medicine, University of Khartoum, Khartoum, SDN; 3 Faculty of Medicine, University of Khartoum, Khartoum, SDN; 4 Pediatrics Department, Sudan Medical Specialization Board, Khartoum, SDN

**Keywords:** bacterial meningitis, clinical training, lumbar puncture, pediatric residents, sudan

## Abstract

Background

Bacterial meningitis (BM) is a life-threatening emergency requiring timely and accurate diagnosis. In Sudan, a country within the meningitis belt, pediatric residents play a key role in BM management, yet no prior studies have evaluated their practices. This study aims to assess diagnostic and management approaches among pediatric residents and identify related challenges.

Methodology

A descriptive cross-sectional study was conducted among pediatric residents registered with the Sudan Medical Specialization Board (SMSB). Data were collected using a validated, self-administered questionnaire distributed via email and WhatsApp. The survey included items on diagnostic methods, lumbar puncture (LP) practices, antibiotic use, training, and follow-up care. Descriptive statistics and Chi-square or Fisher's exact tests were used to analyze associations, with a significance level set at *P *< 0.05.

Results

Of 283 respondents, 249 (88%) were female, and 158 (56%) were in their final residency year. BM diagnosis was confirmed in 106 (37%) cases based on cerebrospinal fluid findings consistent with BM-elevated white cell count, low glucose, and high protein - rather than culture-proven disease, while 40% relied on clinical and basic lab criteria. LP was performed by 212 (75%) of residents, but 168 (59%) reported patient or family refusal as a primary barrier. Only 73 (26%) documented consent/refusal. While 243 (86%) were trained to interpret LP results, just 157 (55%) received training on obtaining consent. Empirical treatment commonly included vancomycin-based regimens (142, 50%) or third-generation cephalosporins (138, 49%). Most residents adhered to a 10- to 14-day intravenous antibiotic regimen (264, 93%), but only 63 (22%) consistently arranged hearing assessments post-treatment.

Conclusions

Findings reveal inconsistent diagnostic practices and significant training and resource gaps among pediatric residents managing BM in Sudan. Addressing these deficiencies may improve diagnostic accuracy, treatment outcomes, and post-discharge care.

## Introduction

Bacterial meningitis (BM) represents a potentially life-threatening medical emergency if not accurately diagnosed and appropriately managed, and it is estimated that more than 1.2 million cases of BM occur globally each year [1]. According to the World Health Organization's Global Disease Burden Data, Sudan is located within the meningitis belt and accounts for approximately 2 out of every 100 cases (15%) [2].

According to WHO guidelines, in both children and adults with suspected acute meningitis who are admitted to an appropriate healthcare facility, empiric intravenous antimicrobial treatment must be administered as promptly as possible. Blood cultures should be obtained at the earliest opportunity, preferably before the initiation of antibiotic therapy [3]. In Sudan, healthcare workers, including residents, often do not consistently adhere to the codes and criteria for the diagnosis of BM, leading to issues of overdiagnosis and excessive prescription of antibiotics; consequently, the diagnosis of BM must be guided by internationally validated clinical prediction rules [1].

Despite the significant role of residents in the diagnosis and management of BM, there is no research addressing their practices in Sudan, creating an evidence gap. This study aims to address this evidence gap by evaluating current diagnostic practices, including LP use, empirical treatment patterns, training exposure, barriers to guideline-based care, and follow-up habits among pediatric residents in Sudan.

## Materials and methods

Study design and participants

We conducted a descriptive, cross-sectional study at the Sudan Medical Specialization Board (SMSB). The SMSB serves as the sole professional training body in the Republic of Sudan, tasked with managing and delivering medical and health specialty programs within the country. The foundational structure of the SMSB comprises specialty councils, which are responsible for implementing all activities related to the training of medical doctors, including the development and review of the curriculum. The Pediatric Council at the SMSB awards clinical MD certificates in pediatrics and child health to trainees who complete a four-year training program. 

Participants were chosen from among SMSB-registered pediatric residents actively practicing in hospitals in Sudan during the study period who encountered a case of suspected BM.

Instrument

Data were collected using an electronic self-administered Google Forms questionnaire that was pretested and validated among 20 residents (Appendix). Residents assigned to this study were contacted individually to complete the questionnaire, which was sent via email or WhatsApp. The questionnaire consisted of a total of 22 items. The initial three fields pertained to residency year, gender, and years of experience as a pediatrician, respectively. The questionnaire included seven questions related to diagnostic practices, three questions about formal training in lumbar puncture (LP) procedures, result interpretation, and consent, six questions concerning management practices, and three questions addressing follow-up and complications.

Sampling and data collection

The study was conducted among 1,054 pediatric residents. The sample size was determined using the finite population formula, which yielded a minimum required sample of 282 residents. This calculation assumed a 95% confidence level, a 5% margin of error, and an estimated response rate of 50%. The survey required an average completion time of five minutes. Data collection occurred from July 2023 to September 2023.

Statistical analysis

Data were collated in Microsoft Office Excel and subsequently imported into R (version 4.4.1) for statistical analysis. R is a free, open-source environment for statistical computing and graphics. Comparative analyses were conducted to examine associations between years of pediatric experience and key practice variables, including diagnostic approach (e.g., use of LP), choice of empirical antibiotics, management decisions following negative cerebrospinal fluid (CSF) results, documentation of consent, and frequency of ordering follow-up assessments such as hearing or vision tests. These categorical variables were compared using Pearson’s Chi-square test or Fisher’s exact test, where appropriate. These tests were selected because the variables were categorical and were chosen based on their clinical relevance and their potential association with residents’ experience, diagnostic practices, and management patterns. A *P*-value of <0.05 was considered statistically significant.

## Results

Among the 283 pediatric residents surveyed, the overwhelming majority were female, representing 249 (88%) of the cohort, while males constituted only 34 (12%). The distribution of clinical experience revealed that over half of the residents (153, 54%) had between two and five years of pediatric practice, whereas 99 (35%) had more than five years, and only 31 (11%) had less than two years of experience. Regarding training level, most participants were in their final year of residency, with 158(56%) identified as fourth-year (final-year) residents. The remaining residents were more evenly distributed across earlier stages: 50 (18%) were third-year residents, 48 (17%) second-year residents, and 25 (8.8%) first-year pediatric residents (Table 1).

**Table 1:** Demographic characteristics of pediatric residents.

Characteristic	*n* (%), *N* = 283
Gender	
Female	249 (88%)
Male	34 (12%)
Years of experience as a pediatrician	
Less than 2 years	32 (11%)
2-5 years	152 (54%)
More than 5 years	99 (35%)
Residency year	
First-year pediatric resident	25 (8.8%)
Second-year resident	49 (17%)
Third-year resident	51 (18%)
Fourth-year (final-year) resident	158 (56%)

When examining whether these demographic factors were associated with diagnostic or management practices, several significant patterns emerged. Residents with more than five years of experience were more likely to have performed LP (240 (85%) vs. 198 (70%) among those with two to five years and 187 (66%) among those with <2 years; *P* = 0.016). Lack of experience as a barrier to performing LP was significantly more common among the least-experienced residents (37 (13%) vs. 3 (1%) in the most experienced group; *P* = 0.007). Training in LP procedures and interpretation also showed statistically significant associations with years of experience (*P* = 0.007 and *P* = 0.036, respectively). Additionally, usual hospital stay duration (*P* = 0.026) and counseling frequency (*P* = 0.029) varied significantly by experience level. Other practices, including diagnostic confirmation methods, reasons for LP non-performance, empirical antibiotic choices, umbrella therapy use, and follow-up arrangements, did not significantly differ across demographic characteristics.

Bacteriological confirmation of suspected BM via LP was achieved in 106 cases (37%), while diagnosis based solely on clinical suspicion accounted for 56 cases (20%). The largest proportion (112, 40%) relied on a combination of standard clinical criteria and basic laboratory investigations, while a small minority (9, 3.2%) diagnosed cases based exclusively on clinical criteria. Notably, 212 (75%) residents reported performing LP themselves during their registrar training. The predominant barrier to performing LP was patient or family refusal, cited by 168 (59%) respondents, followed by limited resources for CSF collection and analysis in 82 (29%), and lack of experience in 18 (6.4%) of cases. Concerns about the low yield or inaccuracy of prior CSF results were less commonly reported (9, 3.2%). Regarding counseling, residents on call were the primary professionals responsible for discussing the procedure in 241 (85%) of instances. Documentation of consent or refusal was inconsistently practiced: only 73 (26%) routinely recorded consent, whereas 156 (55%) did not document consent/refusal (Table 2).

**Table 2:** Practices in diagnosis and lumbar puncture usage for suspected bacterial meningitis. LP, lumbar puncture; CSF, cerebrospinal fluid

Characteristic	*n* (%), *N *= 283
Diagnosis confirmation method	
Bacteriological confirmation using LP	106 (37%)
The patient was diagnosed based on clinical suspicion only	56 (20%)
Standard clinical criteria and basic laboratory investigations	112 (40%)
Standard clinical criteria only	9 (3.2%)
Performed LP as a registrar	212 (75%)
Common reasons for not performing LP	
Patient or family refusal	168 (59%)
Lack of experience to perform LP	18 (6.4%)
Lack of resources to obtain and analyze CSF in the health facility where you work	82 (29%)
The often inaccurate results or low yield of CSF samples previously obtained from other patients	9 (3.2%)
Other	13 (4.6%)
Primary counselor regarding LP	
The resident on-call	241 (85%)
The medical officer on-call	7 (2.5%)
The specialist on-call	35 (12%)
Practice of documenting consent/refusal for LP	
Yes	73 (26%)
No	156 (55%)
Only if the family approved the procedure	13 (4.6%)
Only if the family refused the procedure	41 (14%)

Formal training related to LP varied across different aspects of the procedure. Specifically, 196 (69%) reported having received training to perform LP during their residency, while a higher proportion (243, 86%) were trained to interpret LP results. In contrast, only 157 (55%) had been formally trained to obtain informed consent for the procedure (Table 3).

**Table 3:** Formal training in lumbar puncture (LP) among pediatric residents.

Characteristic	*n* (%), *N* = 283
Trained to obtain informed consent during residency	157 (55%)
Trained to perform LP during residency	196 (69%)
Trained to interpret LP results during residency	243 (86%)

Access to CSF analysis kits in local hospital laboratories was limited, with only 108 (38%) reporting availability, while 156 (55%) indicated the kits were not accessible, and 19 (6.7%) were uncertain. In managing suspected BM with a negative CSF result, clinical approaches varied: 118 (42%) maintained the diagnosis and continued full inpatient antibiotic treatment, 110 (39%) discontinued antibiotics, assuming an alternative diagnosis, and 55 (19%) opted for observation off antibiotics before making further decisions. Empirical antibiotic therapy predominantly involved either combination regimens including vancomycin (142, 50%) or third-generation cephalosporins alone (ceftriaxone) (138, 49%). The practice of initiating broad-spectrum umbrella therapy - comprising antibiotics, antivirals, and antimalarials - was common, with 149 (53%) usually and 33 (12%) always applying this approach. When patients showed no clinical improvement, nearly half of the residents, 130 (46%), escalated antibiotic treatment empirically, while 80 (28%) pursued further diagnostic evaluation through LP or neuroimaging, and 60 (21%) repeated blood workups, including CBC and CRP. Only a small fraction, 13 (4.6%), completed the full antibiotic course before re-evaluation (Table 4).

**Table 4:** Laboratory access and initial management of suspected bacterial meningitis. LP, lumbar puncture; CBC, complete blood count; CRP, C-reactive protein

Characteristic	*n* (%), *N* = 283
Availability of the CSF analysis kit in the local hospital laboratory	
I don't know	19 (6.7%)
No	156 (55%)
Yes	108 (38%)
Management approach for a negative CSF result in suspected bacterial meningitis	
Observe the patient off antibiotics, then decide accordingly	55 (19%)
This is not bacterial meningitis; you would stop antibiotics and look for other causes of the patient's condition	110 (39%)
This is still bacterial meningitis, and the patient will stay admitted to receive the full course of treatment	118 (42%)
Empirical antibiotic choice for suspected bacterial meningitis	
Combination antibiotics, including vancomycin	142 (50%)
Other	3 (1.1%)
Third-generation cephalosporins (ceftriaxone)	138 (49%)
Frequency of initiating umbrella therapy (antibiotics + antiviral + antimalarial)	
Always	33 (12%)
Never	12 (4.2%)
Rarely	89 (31%)
Usually	149 (53%)
Next step when no clinical improvement in bacterial meningitis	
Complete the full course of antibiotics and then re-evaluate	13 (4.6%)
Perform LP if not already done and/or neuroimaging	80 (28%)
Redo blood work-up: CBC, CRP	60 (21%)
Upgrade antibiotics empirically	130 (46%)

The typical hospital stay for patients with suspected or confirmed BM was predominantly 10-14 days of intravenous antibiotic therapy before discharge, as reported by 264 (93%) of respondents. A smaller proportion (12, 4.2%) discharged after up to seven days with a planned transition to oral antibiotics, while 7 (2.5%) discharged only after clinical improvement before switching to oral therapy. Counseling practices about potential complications were consistently emphasized, with 207 (73%) always and 59 (21%) often providing such counseling, indicating a strong focus on patient and family education. Follow-up arrangements were similarly prioritized, as 159 (56%) scheduled multiple visits, 99 (35%) arranged a single follow-up, and only 25 (8.8%) reported no follow-up was usually required. Audiological monitoring was requested less consistently: 63 (22%) always and 78 (28%) often ordered hearing assessments, but 108 (38%) did so rarely and 34 (12%) never, reflecting variability in adherence to screening guidelines. Vision assessments were even less frequently requested, with only 40 (14%) always and 75 (27%) often arranging evaluations, while 115 (41%) rarely and 53 (19%) never did (Table 5).

**Table 5:** Outcomes, counseling practices, and follow-up in bacterial meningitis care.

Characteristic	*n* (%), *N* = 283
Usual hospital stay duration for suspected or confirmed bacterial meningitis	
10-14 days on intravenous antibiotics, then get discharged	264 (93%)
Stay for up to 7 days, then discharged on oral antibiotics	12 (4.2%)
Till clinical improvement is made, then discharged on oral antibiotics	7 (2.5%)
Frequency of counseling patients or families about complications	
Always	207 (73%)
Often	59 (21%)
Rarely	17 (6.0%)
Never	0 (0%)
Frequency of follow-up visit arrangements	
More than once	159 (56%)
No follow-up visit is usually required	25 (8.8%)
Once	99 (35%)
Frequency of requesting a hearing assessment in bacterial meningitis patients	
Always	63 (22%)
Often	78 (28%)
Rarely	108 (38%)
Never	34 (12%)
Frequency of requesting vision assessment in bacterial meningitis patients	
Always	40 (14%)
Often	75 (27%)
Rarely	115 (41%)
Never	53 (19%)

Diagnostic approaches and LP practices among pediatric residents showed some variation when stratified by years of experience. Bacteriological confirmation via LP was reported in 106 cases (37%) overall, with a nonsignificant trend toward higher confirmation rates among those with more than five years of experience (44, 44%) compared with those with two to five years (50, 33%) and those with less than 2 years of experience (12, 38%) (*P* = 0.2). Clinical suspicion alone was less commonly relied upon among the most experienced group (13, 13%) compared with the intermediate (37, 24%) and least experienced groups (6, 19%). The majority (212, 75%) performed LPs as registrars; however, this practice was significantly more common among those with over five years of experience (84, 85%) compared with those with two to five years (107, 70%) and under two years of experience (21, 66%) (*P* = 0.016).

Reasons for not performing LP were largely consistent across groups, with patient or family refusal cited in 168 cases (59%) overall and no significant differences by level of experience (*P* = 0.4). However, lack of experience as a barrier was significantly more common among residents with less than two years of experience (4, 13%) compared with those with more than five years of experience (1, 1%) (*P* = 0.007). Resource limitations and concerns about CSF sample accuracy did not differ significantly by level of experience.

Counseling about LP was predominantly delivered by the resident on call (241, 85%) across all groups, with no significant differences observed. Documentation of consent or refusal for LP was generally poor, with only 37 (26%) overall consistently recording it, and this did not differ significantly by experience level (*P* = 0.7) (Table 6).

**Table 6:** Diagnostic approaches and lumbar puncture (LP) practices stratified by years of pediatric experience.

Characteristic	Overall, *n* (%), *N* = 283	2-5 years, *n* (%), *N* = 152	Less than 2 years, *n* (%), *N* = 32	More than 5 years, *n* (%), *N* = 99	*P*-value
Diagnosis confirmation method					0.2
Bacteriological confirmation using LP	106 (37%)	50 (33%)	12 (38%)	44 (44%)	
The patient was diagnosed based on clinical suspicion only	56 (20%)	37 (24%)	6 (19%)	13 (13%)	
Standard clinical criteria and basic laboratory investigations	112 (40%)	59 (39%)	12 (38%)	41 (41%)	
Standard clinical criteria only	9 (3.2%)	6(3.9%)	2(6.3%)	1 (1.0%)	
Performed LP as a registrar	212(75%)	107(70%)	21(66%)	84(85%)	0.016
Common reasons for not performing LP					
Patient or family refusal	168(59%)	87 (57%)	17 (53%)	64 (65%)	0.4
Lack of experience to perform LP	18 (6.4%)	13(8.6%)	4 (13%)	1 (1.0%)	0.007
Lack of resources to obtain and analyze CSF in the health facility where you work	82 (29%)	44 (29%)	10 (31%)	28 (28%)	>0.9
The often inaccurate results or low yield of CSF samples previously obtained from other patients	9 (3.2%)	7 (4.6%)	1 (3.1%)	1 (1.0%)	0.2
Other	13 (4.6%)	8 (5.3%)	0 (0%)	5 (5.1%)	0.5
Primary counselor regarding LP					>0.9
The resident on-call	241(85%)	128(84%)	27(84%)	86 (87%)	
The medical officer on-call	7 (2.5%)	4 (2.6%)	1 (3.1%)	2 (2.0%)	
The specialist on-call	35 (12%)	20 (13%)	4 (13%)	11 (11%)	
Practice of documenting consent or refusal for LP					0.7
Yes	73(26%)	44(29%)	5 (16%)	24(24%)	
No	156(55%)	81 (53%)	22(69%)	53 (54%)	
Only if the family approved the procedure	13 (4.6%)	6 (3.9%)	1 (3.1%)	6 (6.1%)	
Only if the family refused the procedure	41 (14%)	21 (14%)	4 (13%)	16 (16%)	

Training in LP skills among pediatric residents varied significantly by years of experience. Overall, 157 (55%) reported receiving training on obtaining informed consent during residency, with a trend toward higher training rates among those with more than five years of experience (63, 64%) compared with those with two to five years of experience (81, 53%) and those with less than two years of experience (13, 41%), approaching but not reaching statistical significance (*P* = 0.054). A more pronounced difference was observed in training to perform LP, with 196 (69%) trained overall and significantly higher training rates among the most experienced group (79, 80%) compared with the intermediate (100, 66%) and least experienced groups (17, 53%) (*P* = 0.007). Similarly, training to interpret LP results was highest among residents with more than five years of experience (91, 92%) compared with those with two to five years (128, 84%) and less than two years of experience (24, 75%) (*P* = 0.036) (Table 7).

**Table 7:** Training in consent, procedure, and interpretation of lumbar puncture (LP) among pediatric residents by years of experience.

Characteristic	Overall, *n* (%), *N* = 283	2-5 years, *n* (%), *N* = 152	Less than 2 years, *n* (%), *N* = 32	More than 5 years, *n* (%), *N* = 99	*P*-value
Trained to obtain informed consent during residency	157 (55%)	81 (53%)	13 (41%)	63 (64%)	0.054
Trained to perform LP during residency	196 (69%)	100 (66%)	17 (53%)	79 (80%)	0.007
Trained to interpret LP results during residency	243 (86%)	128 (84%)	24 (75%)	91 (92%)	0.036

Table 8 presents data on laboratory access, empirical treatment choices, and management strategies when treatment fails in suspected BM, stratified by pediatricians’ years of experience. The approach to managing negative CSF results was comparable between groups: about 118 (42%) considered ongoing BM requiring full treatment, 110 (39%) would stop antibiotics and explore other diagnoses, and 55 (19%) would observe off antibiotics before deciding, without significant variation (*P* = 0.5).

Empirical antibiotic regimens also did not significantly differ by experience, with roughly half (142, 50%) favoring combination therapy including vancomycin and nearly an equal proportion (138, 49%) using third-generation cephalosporins alone (ceftriaxone) (*P* = 0.2). The frequency of initiating umbrella therapy - covering antibiotics, antivirals, and antimalarials - was consistent among groups, with the majority usually or always employing this approach (*P* > 0.9).

When clinical improvement was not observed, the most common next step was empiric antibiotic escalation (130, 46%), followed by performing LP or neuroimaging (80, 28%) and repeating blood work (60, 21%). These strategies were similarly applied regardless of pediatric experience (*P* = 0.7). Notably, residents with less than two years of experience were more likely (13, 41%) to perform LP or neuroimaging at this stage compared to other groups, although this did not reach statistical significance.

**Table 8:** Laboratory access, empirical treatment, and response to treatment failure in suspected bacterial meningitis by years of pediatric experience. LP, lumbar puncture; CBC, complete blood count; CRP, C-reactive protein

Characteristic	Overall, *n* (%), *N* = 283	2-5 years, *n* (%), *N* = 152	Less than 2 years, *n* (%), *N* = 32	More than 5 years, *n* (%), *N* ​​​​​​​ = 99	*P*-value
Management approach for a negative CSF result in suspected bacterial meningitis					0.5
Observe the patient off antibiotics, then decide accordingly	55 (19%)	25 (16%)	5 (16%)	25 (25%)	
This is not bacterial meningitis; you would stop antibiotics and look for other causes of the patient's condition	110 (39%)	62 (41%)	14 (44%)	34 (34%)	
This is still bacterial meningitis, and the patient will stay admitted to receive the full course of treatment	118 (42%)	65 (43%)	13 (41%)	40 (40%)	
Empirical antibiotic choice for suspected bacterial meningitis					0.2
Combination antibiotics, including vancomycin	142 (50%)	79 (52%)	11 (34%)	52 (53%)	
Other	3 (1.1%)	3 (2.0%)	0 (0%)	0 (0%)	
Third-generation cephalosporins (ceftriaxone)	138 (49%)	70 (46%)	21 (66%)	47 (47%)	
Frequency of initiating umbrella therapy (antibiotics + antiviral + antimalarial)					>0.9
Always	33 (12%)	19 (13%)	4 (13%)	10 (10%)	
Never	12 (4.2%)	7 (4.6%)	1 (3.1%)	4 (4.0%)	
Rarely	89 (31%)	47 (31%)	9 (28%)	33 (33%)	
Usually	149 (53%)	79 (52%)	18 (56%)	52 (53%)	
Next step when no clinical improvement in bacterial meningitis					0.7
Complete the full course of antibiotics and then re-evaluate	13 (4.6%)	7 (4.6%)	2 (6.3%)	4 (4.0%)	
Perform LP if not already done and/or neuroimaging	80 (28%)	39 (26%)	13 (41%)	28 (28%)	
Redo blood work-up: CBC, CRP	60 (21%)	31 (20%)	6 (19%)	23 (23%)	
Upgrade antibiotics empirically	130 (46%)	75 (49%)	11 (34%)	44 (44%)	

Table 9 outlines inpatient care practices, counseling frequency, follow-up arrangements, and sensory assessment habits in BM management, stratified by pediatricians’ years of experience.

**Table 9:** Inpatient care, counseling, follow-up, and sensory assessment practices in bacterial meningitis by years of pediatric experience.

Characteristic	Overall, *n* (%), *N* = 283	2-5 years, *n* (%), *N* = 152	Less than 2 years, *n* (%), *N* ​​​​​​​ = 32	More than 5 years, *n* (%), *N* ​​​​​​​ = 99	*P*-value
Usual hospital stay duration for suspected or confirmed bacterial meningitis					0.026
10-14 days on intravenous antibiotics, then get discharged	264 (93%)	140 (92%)	28 (88%)	96 (97%)	
Stay for up to 7 days, then discharged on oral antibiotics	12 (4.2%)	10 (6.6%)	1 (3.1%)	1 (1.0%)	
Till clinical improvement is made, then discharged on oral antibiotics	7 (2.5%)	2 (1.3%)	3 (9.4%)	2 (2.0%)	
Frequency of counseling patients or families about complications					0.029
Always	207 (73%)	110 (72%)	22 (69%)	75 (76%)	
Often	59 (21%)	27 (18%)	10 (31%)	22 (22%)	
Rarely	17 (6.0%)	15 (9.9%)	0 (0%)	2 (2.0%)	
Frequency of follow-up visit arrangements					0.059
More than once	159 (56%)	80 (53%)	13 (41%)	66 (67%)	
No follow-up visit is usually required	25 (8.8%)	16 (11%)	3 (9.4%)	6 (6.1%)	
Once	99 (35%)	56 (37%)	16 (50%)	27 (27%)	
Frequency of requesting a hearing assessment in bacterial meningitis patients					0.3
Always	63 (22%)	31 (20%)	6 (19%)	26 (26%)	
Never	34 (12%)	24(16%)	4 (13%)	6 (6.1%)	
Often	78 (28%)	38 (25%)	9 (28%)	31 (31%)	
Rarely	108 (38%)	59 (39%)	13 (41%)	36 (36%)	
Frequency of requesting vision assessment in bacterial meningitis patients					0.5
Always	40 (14%)	22 (14%)	3 (9.4%)	15 (15%)	
Never	53 (19%)	35 (23%)	6 (19%)	12 (12%)	
Often	75 (27%)	36 (24%)	10 (31%)	29 (29%)	
Rarely	115 (41%)	59 (39%)	13 (41%)	43 (43%)	

There is a statistically significant difference in the usual hospital stay duration (*P* = 0.026). Most pediatricians (264, 93% overall) follow the standard practice of 10-14 days of intravenous antibiotics before discharge, with the highest adherence among those with more than five years of experience (96, 97%). Those with less than two years of experience are slightly less likely (28, 88%) to follow this protocol and more likely (3, 9.4%) to discharge patients once clinical improvement is made and switch to oral antibiotics.

Counseling frequency also varies significantly (*P* = 0.029). The majority “always” counsel patients or families about potential complications (207, 73% overall), with more experienced pediatricians (75, 76%) slightly more consistent in doing so compared to those with less than two years (22, 69%). Pediatricians with less than two years of experience show a higher tendency to counsel “often” (10, 31%), and none reported “rarely” counseling, unlike other groups.

Follow-up visits show a borderline difference (*P* = 0.059). More experienced pediatricians tend to arrange multiple follow-up visits (66, 67%), whereas those with less than two years of experience more often schedule only one visit (16, 50%).

Requesting hearing assessments after meningitis was fairly consistent across groups, with no significant difference (*P* = 0.3). Overall, 63 (22%) always requested it, 78 (28%) often, 108 (38%) rarely, and 34 (12%) never, with residents having over five years of experience being marginally more likely to “always” or “often” request it.

Similarly, vision assessment requests showed no significant variation (*P* = 0.5), with most pediatricians rarely or often requesting vision tests. The proportion of those “always” requesting vision assessments remained low across all experience levels (40, approximately 14%).

## Discussion

This study provides insights into the practices, challenges, and training gaps among pediatric residents in Sudan regarding BM diagnosis and management. The findings reveal substantial variability in diagnostic approaches, management protocols, and follow-up practices.

Our primary aim was to assess pediatric residents' practices regarding BM. The results indicate that while residents use various diagnostic methods, there is inconsistency in their approach. Only 37% confirmed BM bacteriologically through LP, while 40% relied on clinical criteria and basic lab investigations. The remaining 20% based their diagnosis solely on clinical suspicion. This variability reflects the challenges of clinical practice in resource-limited settings, where clinicians often depend on clinical features and basic laboratory data [4]. 

The study significantly underscores barriers to optimal management of BM in Sudan, particularly in relation to LP performance. LP is crucial in young children for detecting meningitis, as in some cases, a microorganism may be seen on CSF microscopy, guiding treatment [5]. A notable 59% of respondents identified patient or family refusal as the principal obstacle, followed by a lack of resources for CSF collection and analysis, cited by 29% of participants. This finding is consistent with broader challenges reported across sub-Saharan Africa, where high rates of LP refusal can significantly compromise the quality of clinical care. These elevated refusal rates prompt several inquiries, including whether the reasons for LP refusal are specific to region, sex, or disease, and whether such refusals are influenced by patients' perceptions or by practitioners' discomfort with the procedure [6]. 

Although most residents reported performing LP during training, only 55% had formal instruction on obtaining consent. This may partially explain both the low documentation rate for consent/refusal (26%) and the high refusal rates from families. Inadequate communication skills may result in families feeling uncertain or fearful about the procedure, reinforcing the need for more robust consent training. Compared to findings from a study conducted in Pakistan, which identified that the primary reasons for parental refusal of LP in children were concerns regarding the potential for limb paralysis and apprehensions about mortality [7]. A study by Narchi et al. found that understanding, perceptions, beliefs, and fears of the potential complications of meningitis were significant factors in parents consenting to the procedure [8].

The empirical antibiotic practices reported in our study align partially with current WHO recommendations. Our finding that 50% of residents used vancomycin-containing combination regimens and 49% used third-generation cephalosporins alone reflects awareness of recommended practices, but reveals implementation gaps. The WHO guidelines strongly recommend ceftriaxone or cefotaxime as first-line agents for BM [9]. The widespread practice of umbrella therapy (including antibiotics, antivirals, and antimalarials) by 65% of respondents suggests defensive prescribing patterns that may reflect diagnostic uncertainty or resource constraints limiting pathogen identification.

The usual hospital stay for suspected or confirmed BM is aligned with standard treatment guidelines. Most residents (93%) reported administering a fixed 10-14-day course of intravenous antibiotics; while consistent, this practice does not reflect pathogen-specific treatment durations and may indicate limited individualization of care. This adherence was compromised by experience level: less experienced residents were significantly more likely to report discharge based solely on clinical improvement and a shift to oral antibiotics. This practice, which risks incomplete treatment and relapse, highlights a specific knowledge-practice gap in discharge criteria among junior residents. In our study, the high rate of LP refusal (59%) likely contributes to the extended antibiotic courses and hospital stays reported by residents [10], thereby increasing the burden on the Sudanese healthcare system.

Regarding follow-up practices, our finding that only 22% of residents always ordered hearing assessments post-meningitis is concerning but consistent with evidence from other African countries. A South African study found that almost 40% of pediatric meningitis survivors were never referred for audiological services, with barriers including non-referral by physicians, insufficient diagnostic equipment, and inadequate numbers of audiologists [11]. This highlights a critical gap in the continuity of care for these vulnerable patients across the region.

The present results mirror trends seen in other low- and middle-income countries, where limited diagnostic capacity and insufficient procedural training hinder guideline adherence. A Moroccan survey reported that 50% of pediatric residents had never performed an LP, and none had received simulation-based training [12]. In neonatal practice, studies have reported high failure rates for LP procedures, with one prospective cohort showing a 38.2% failure rate that was not associated with operator experience or other evaluated factors [13]. Embedding such modules in Sudan’s pediatric residency curriculum could reduce LP refusal rates and improve diagnostic accuracy. 

The study's strengths include being one of the first to explore these themes in Sudan, adding valuable local context to a global health issue. However, limitations exist. Reliance on self-reported data may introduce bias, reflecting idealized behavior rather than actual practice. Additionally, the cross-sectional design cannot account for seasonal or institutional variability. The lack of standardized BM management guidelines across hospitals may have influenced residents’ perceptions and self-reported practices, contributing to variability in responses. A further limitation is the potential for non-response bias and the demographic skew of the respondents. With an overwhelming majority being female and over half in their final residency year, the findings may not be fully generalizable to the broader population of pediatric residents in Sudan, particularly those who are earlier in their training or are male. Future research should include direct observation of clinical encounters, qualitative interviews with residents and families, and evaluations of patient outcomes for deeper insight.

## Conclusions

This study highlights variability in pediatric residents' practices for BM in Sudan. Most residents were familiar with LP and antibiotic protocols, but gaps in training, consent documentation, and post-discharge follow-up, especially sensory assessments, were noted. High LP refusal rates and limited access to cerebrospinal fluid analysis indicate systemic barriers. These findings underscore the need for standardized training, improved resources, and enhanced clinical protocols for consistent, evidence-based care.

## Appendices

Appendix

**Table 10:** Questionnaire on the diagnosis and management of bacterial meningitis by pediatric residents in Sudan.

No.	Section	Question	Response Options
1	Demographics	Residency year	R1 R2 R3 R4
2	Demographics	Gender	Male Female
3	Experience	Years of experience as a pediatrician	<2 years 2–5 years >5 years
4	Diagnostic Practice	Have you diagnosed bacterial meningitis in your practice?	Yes No
5	Diagnostic Practice	How was the diagnosis confirmed?	Clinical criteria only LP with bacteriological confirmation Clinical criteria + basic labs (WBC, CRP) Clinical suspicion only
6	Diagnostic Practice	Have you performed lumbar puncture (LP) as a registrar?	Yes No
7	Diagnostic Practice	If LP was not performed, what was the usual reason?	Family refusal Lack of experience Lack of resources Low yield/inaccurate CSF Other
8	Diagnostic Practice	In your daily practice, Who usually counsel patients/families about LP?	Specialist Medical officer Me (Resident on call) House officer
9	Diagnostic Practice	Do you obtain written consent or document refusal for LP?	Yes No Only if approved Only if refused
10	Formal Training	Taking informed consent for LP formal training	Yes No
11	Formal Training	Perform LP formal training	Yes No
12	Formal Training	Interpret LP results training	Yes No
13	Diagnostic Practice	Is a CSF analysis kit available in your hospital laboratory?	Yes No Don’t know
14	Management Practice	If CSF results are negative for bacterial meningitis, what is your management plan?	Consider it bacterial meningitis and continue full treatment Consider it not bacterial meningitis, stop antibiotics, investigate other causes Observe patient off antibiotics and decide based on progress
15	Management Practice	In suspected bacterial meningitis, what is your usual empirical antibiotic?	Third-generation cephalosporin Cephalosporin + vancomycin Meropenem Other
16	Management Practice	How often do you start “umbrella treatment” (antibiotic + antiviral + antimalarial) empirically?	Always Usually Rarely Never
17	Management Practice	If a patient with bacterial meningitis does not show initial improvement, what is your next step?	Upgrade antibiotics empirically Redo blood work-up (CBC, CRP) Complete full antibiotic course then reassess Perform LP (if not done) and/or neuroimaging
18	Management Practice	How long do patients with suspected or confirmed bacterial meningitis usually stay admitted?	10-14 days on intravenous antibiotics Until clinical improvement, then discharged on oral antibiotics Up to 7 days, then discharged on oral antibiotics Until patient becomes afebrile
19	Management Practice	How often do you counsel patients/families about possible complications?	Always Often Rarely Never
20	Follow-up and complications	How often is follow-up arranged for bacterial meningitis patients?	Once More than once No follow-up
21	Follow-up and complications	How often do you request hearing assessment for bacterial meningitis patients?	Always Often Rarely Never
22	Follow-up and complications	How often do you request vision assessment for these patients?	Always Often Rarely Never
